# Supplementary material for: Are carbon emissions trading and green financial instruments synergistic? -Comprehensive quantitative research based on content analysis
Source: PLoS One. 2024 Mar 7;19(3):e0298601. doi: 10.1371/journal.pone.0298601 (PMC10919601; doi:10.1371/journal.pone.0298601)
Supplement: S1 Data — (ZIP) [file pone.0298601.s001.zip › Data/地级市Z_F工作报告.zip!/╔╜╬≈/┤≤═1⁄4╩╨/└┤╫╘╣1⁄2╓┌║┼í╛┬φ┐╦╩2╛▌═°í┐_2017.docx]

各位代表：

现在，我代表市人民政府，向大会报告工作，请予审议，并请市政协委员和其他列席人员提出意见。

一、过去五年和2016年重点工作回顾

过去五年，面对复杂严峻的经济形势和艰巨繁重的改革发展稳定任务，全市上下认真贯彻党的十八大，十八届三中、四中、五中、六中全会和习近平总书记系列重要讲话精神，全面落实省委、省政府和市委决策部署，主动适应经济新常态，积极探索转型新路径，稳增长、促改革、调结构、惠民生、防风险，攻坚克难，砥砺前行，取得了令人瞩目的成效。

五年来，我们专注发展、主动作为，经济保持平稳增长。地区生产总值跨越千亿大关，达到1025．3亿元，年均增长7．1％。固定资产投资达到1212．8亿元，实现五年翻番。社会消费品零售总额达到609亿元，年均增长11．3％。

五年来，我们聚焦产业、调转结合，转型步伐明显加快。设施农业突破27万亩，规模养殖场达到920个，农业龙头企业达到176家，粮食产量实现七连增，顺利跻身全国北方农牧交错带核心示范区。煤电等传统产业先进产能稳步提升。光电、风电装机容量达到320万千瓦，占全省的三分之一，成为国家新能源示范城市。现代医药、装备制造等优势产业规模扩大。以文化旅游为重点的现代服务业蓬勃发展，成功举办国际太阳能十项全能大赛、世界养生大会、全省旅游发展大会等重大赛事和节庆活动，旅游总收入达到363．3亿元，年均增长22％。三次产业结构由5．3∶50．7∶44调整为5．8∶36．5∶57．7，第三产业比重逐年提升。

五年来，我们深化改革、致力创新，发展动力不断增强。供给侧结构性改革和转型综改步伐加快，关键环节和重点领域改革深入推进，行政管理改革成效明显。创新元素不断增多，新增各类市场主体9．8万户。大众创业、万众创新势头良好。全力对接京津冀，主动融入环渤海，积极参与乌大张，区域战略合作取得重大突破。大张、大西高铁开工建设，高速公路实现县县通，成为全国性综合交通枢纽城市（货运类）、区域流通节点城市、国家电商与物流快递协同发展试点城市。

五年来，我们注重统筹、建管并重，城乡一体协调发展。城市总体规划修编完成，五规合一持续推进，城市发展框架基本定型。古城墙合拢，护城河贯通，明堂遗址公园对外开放，代王府主体工程完工。博物馆、行政中心投入使用。大县城建设步伐加快，城镇化率达到62％。智慧城市建设积极推进，城市管理和服务水平稳步提升。

五年来，我们标本兼治、保护生态，环境质量显著改善。新增城市绿化面积976．5万平方米，完成营造林190．9万亩，成功创建国家园林城市。严格落实“气十条”“水十条”“土十条”，8条主要河流断面水质止降回升，完成水土保持治理面积168万亩，二级以上良好天数连续四年全省第一，“大同蓝”已经成为响亮的金字招牌。

五年来，我们尊崇民意、尽心竭力，人民福祉持续提高。累计投入财政资金1014．4亿元，全力发展民生事业。养老、医疗保险实现全覆盖，社会保障体系不断完善。就业形势基本稳定。实施整村推进项目212个，减贫人口18万人。农民收入增幅连续五年高于城市居民。办学条件显著改善，义务教育均衡化迈出重要步伐，教育事业协调推进。县级公立医院改革全面铺开，分级诊疗制度付诸实施，医疗联合体建设实现两个全覆盖。解决近30万人饮水安全问题。开工保障性住房28．7万套、建成21．4万套，开工老旧小区综合整治858．5万平方米、完成627．8万平方米，城乡住房条件发生根本变化。民生实事有诺必践，安全生产常抓不懈，社会局面和谐稳定。

五年来，我们履职尽责、提高效能，政府自身建设得到加强。认真开展党的群众路线教育实践活动、三严三实、两学一做等专题学习教育，牢固确立四个意识，从严落实主体责任，坚持践行八项规定，严格遵守约法三章，坚决保持清正廉洁。主动接受人大法律监督、政协民主监督以及各方面监督，办理人大代表建议639件、政协委员提案1214件。推进科学决策、民主决策、依法决策。深化行政审批制度改革，12345政府服务热线快捷畅通，13710政府工作机制基本建立，政府购买服务领域逐步延伸，便民惠民服务体系趋于完善。连续两年打响打胜以“马上就办、真抓实干”为主旨的冬季行动，成为我们转变作风、提高效率的重要创举和有效载体。

各位代表，回顾过去的五年，变化卓著，有目共睹，来之不易；反观刚刚过去的一年，我们同心同力，直面挑战，积极有为，千方百计稳增长，围绕转型促改革，全力以赴谋发展，克服困难保民生，赢得了十三五较好开局。

过去的一年，我们经受了“L”型经济突变，奋力实现经济运行止跌趋稳。面对二季度工业经济断崖式下跌的严峻态势，多措并举，精准发力，积极帮扶企业轻装上阵、扩大产销、降低成本、挖潜增效。非煤工业实现较快增长，服务业和建筑业对经济增长贡献率进一步增大。GDP增长1％，实现了下半年好于上半年、全年转负为正。

过去的一年，我们落实了中央和省市重大决策部署，着力化解体制性、结构性、素质性矛盾。不折不扣落实“三去一降一补”任务，压减煤炭产量2976万吨，占全省任务的五分之一，占全国任务的八分之一。争取政策性贷款140亿元用于棚改货币化安置，深度消化商品房库存。争取置换债券资金化解政府债务，减少财政负担3亿元。争取直供电交易37．1亿千瓦时，惠及企业20多家。落实减税降费政策，为企业减负63亿元。启动开发区、国企国资、同煤三供一业、旅游管理体制等一系列重点改革。把脱贫攻坚作为第一责任，力度空前补短板，159个村有序退出、4．9万人脱贫。大力处置债务拖欠、社会保障、房屋权属等一大批复杂遗留问题，促进了社会和谐。积极回应群众关切，办理民生实事12件。

过去的一年，我们狠抓了一批重大工程建设，倾力增强发展后劲。加快城乡基础设施建设，新建续建城市道路60．5公里，新建改造供热管网257公里、供水管网104公里、供气管网126公里，北环桥、武定东桥、清远南北桥建成通车。加快产业项目建设，全国最大的光伏先进技术示范基地一期100万千瓦项目并网发电，全球首个10万千瓦熊猫光伏电站奠基开工，100万千瓦高效单晶光伏组件领跑项目签约；同煤塔山二期2×66万千瓦机组、山柴煤层气发电机组研发项目试运行，国电湖东2×100万千瓦项目取得路条；晋商联盟古城东北隅开发项目落地，万达广场主体完工，大同—太原旅游专列开通运营，方特欢乐世界主题公园开园迎客，古都灯会开创了大同文化旅游冬令时。

过去的一年，我们促进了社会事业协调发展，全力提升共建共享水平。城乡常住居民人均可支配收入分别达到26273元和8217元，分别增长6．1％和6．6％。公共财政预算收入完成88．9亿元，增速高出全省1．4个百分点，在财政收入增长明显放缓的情况下，既实现了保基本、保运转、保支出，又支持了重大工程和项目建设。城镇新增就业4．28万人。居民消费价格指数涨幅1．1％，比全国低0．9个百分点。实施教育、卫生等多项重要工程，公共服务水平持续提升。启动御河、十里河等流域生态治理重大项目，坚持控煤、治污、管车、降尘四管齐下，二级以上良好天数达到314天。其他约束性指标任务全部完成。有效化解各类信访案件。工矿商贸企业事故起数和死亡人数实现双下降。深入开展百日严打整治专项行动，有力震慑了违法犯罪。

过去的一年，我们提振了干部队伍精气神，强力促进创新驱动、转型升级。积极参加“读党书、论责任、抓落实”干部专题培训，全市人心思上、人心思进，广大干部的责任意识大幅提升，干事创业热情日益高涨，一个中心、三个大同发展目标和136发展战略深入人心，创新驱动、转型升级成为发展的最强音。全市上下内联外引，谋划布局了总投资690亿元的双环行动项目、总投资2000多亿元的16字项目，这既是形势所迫，也是转型所需，更是合力所为。我们有理由相信，这些项目、这股热潮、这种精气神，必将催生大同全面振兴的春天。

上述成绩的取得，是在市委的正确领导下，在市人大、市政协的监督支持下，全市干部群众上下同心、奋力拼搏的结果，也饱含着历任政府班子团结协作、奋发图强的担当情怀。在此，我代表市人民政府，向全市人民，向全体人大代表、政协委员，向各民主党派、工商联、各人民团体，向离退休老同志和社会各界人士，向驻同部队、武警官兵和公安干警，向所有关心、支持和参与大同改革发展的同志们、朋友们，致以最崇高的敬意，表示最衷心的感谢！

我们必须清醒地看到，经济社会发展中还存在着困难和问题，经济层面，一煤独大、一企独大的矛盾没有根本改观，经济发展新旧动能转换乏力，确保经济运行回归合理区间还需持续努力。社会层面，社会管理的理念、制度、方式亟待创新，各种社会组织的作用尚未充分发挥。民生层面，各级财政保障能力有限，民生改善速度和水平短期内难以满足群众需求，一些领域的公共服务质量提高不快，距离全面小康还需加倍努力。政府工作层面，政府职能转变有待不断深化，懒政怠政现象依然不同程度存在，等等。对此，我们一定要高度重视、认真解决。

二、新一届政府工作建议

未来五年，是大同落实发展新理念、找准发展新方位、开启发展新征程的五年，使命光荣，责任重大。我们必须按照省委“一个指引、两手硬”重大思路和要求，认真贯彻落实省市党代会的战略部署，抢抓三个倒逼的历史机遇，忠诚担当，迎难而上，主动转型、创新转型、深度转型、全面转型。

根据市第十五次党代会提出的奋斗目标，建议在全面完成十三五规划的基础上，今后五年大同经济社会发展的主要预期目标是：经济保持中高速增长，多元化中高端现代产业体系基本构建；改革、开放、创新实现重大突破，开放型经济水平大幅提升；绿色发展理念广泛认同，生态文明建设成果丰硕；民生和社会事业全面进步，与全国全省同步建成全面小康社会。

围绕上述目标，重点推动四个转型：

——围绕打造区域性中心城市，加快推动城市转型。立足区位优势，抢抓中央全面加快城镇化和非首都功能疏解的政策机遇，格局做大，布局做优，产业做强，声誉做响，着力打造晋北城市群和晋冀蒙金三角的龙头城市。一是向全方位全国性综合交通枢纽城市转变。巩固以煤炭为主的货运类全国性综合交通枢纽城市地位，加大力度，加快速度，持续推进客运、货运全功能通道建设。大张、大西、大乌高铁建成通车组成大动脉，公路提标升级联成全网络，航线拓展延伸形成强辐射，轻轨通联城市四方构建新框架，基本形成畅通发达的立体式交通格局。同步加快输电、输气等国家重大能源输送通道工程建设，全面提升城市战略地位。二是向生产要素集聚城市转变。冲破资源型城市束缚，树立全新资源观，推动全要素快速成长。土地梯次储备，供应机制灵活；金融体系拓展，筹集渠道多元；技术不断创新，应用领域广泛；人才规模使用，构成发展中坚；管理科学有效，企业类聚集群，基本形成要素集成、流转集中、利用集约的发展洼地。三是向现代商贸城市转变。改造传统商贸流通产业，大力建设新基地新设施，发展新业态新模式。华阳国际陆港、晋北国际物流园等综合体建成运营，主要工业品、特色农产品市场体系基本健全，实体销售与互联网经济互促共赢，灵敏便捷高效的现代物流机制基本形成，建成国家电商与物流快递协同发展城市。四是向全域旅游城市转变。迎接大众旅游时代的到来，尽快实现景点景区旅游城市华丽转身，着力打造以大同都市为核心，以县区为支撑，以特色城镇、历史古镇、传统村落为补充，建设古都风情魅力独具、新区时代风尚凸显、县区地域特色浓郁、村镇历史记忆真切的全域文化旅游城市。大同古城、云冈石窟、北岳恒山力争成为世界级文化旅游胜地，火山群、古长城、北魏皇陵三大新景点初具规模，浑源、灵丘、大同县建成国家全域旅游示范区，生态游、商务游、乡村游以及运动娱乐、养生养老等新业态蓬勃发展。文化旅游体制机制全面创新，旅游市场发育成熟，基本建成在全国乃至世界堪称一流的旅游目的地城市。

——围绕建设富裕大同，加快推动经济转型。坚持创新驱动、投资拉动、改革推动、县区联动，着力打造转型升级项目群，厚植实体经济发展新优势，形成新旧动能转换加速度，推动产业迈向中高端，发展保持中高速。一是发展重点由煤炭支柱型向多元支撑型转变。以优化升级能源产业、着力培育替代产业、发展壮大新兴产业、做大做强文化旅游产业为路径，加快产业优化升级。能源产业方面，煤炭产业主攻千万吨级现代化矿井，地方煤矿落后产能基本淘汰；力争火电装机突破1000万千瓦，光电、风电装机850万千瓦，抽水蓄能装机150万千瓦。替代产业方面，以轨道交通、煤机制造、新能源汽车制造、航空制造为主的装备制造产业实现规模扩张和提质增效，以煤炭下游产品为主的现代煤化工产业链条初步形成，以石墨、玄武岩、花岗岩为主的新材料产业渐成规模。新兴产业和旅游产业方面，数字经济加速培育，以康养、物流、文化旅游为主的现代服务业集聚发展，经济朝着低碳、绿色、循环方向稳健迈进。非煤工业占工业比重稳定在50％以上，现代服务业增加值占GDP比重突破20％。二是发展动力由要素驱动型向创新驱动型转变。“双创”服务体系建立健全，各类主体各展其长，线上线下良性互动。一批重大科技创新项目顺利实施。绿色金融改革创新试验区、科技创新基地和京津冀区域协调发展创新高地基本建成。市场环境进一步优化，人才智库发挥积极作用，迈上集约创新发展之路。三是发展方式由规模速度型向质量效益型转变。落实GDP“三要三不要”要求，资源消耗型、粗放增长型经济逐步萎缩，重工业特征逐步淡化，产业科技层次逐步提升，资源节约型、环境友好型产业成为主要经济版块，绿色门槛把得住，生态红线守得紧，增长速度稳中求快，综合效益不断提升，努力成为资源型城市经济成功转型的“大同样本”。

——围绕建设美丽大同，加快推动生态转型。完善生态补偿机制和资源环境监测预警机制，推行绿色生产生活方式，涵养绿色气质，提升生态颜值，全面展示山水人文共生、生态绿色共享的美丽形象。一是推进生产方式清洁化。培育壮大节能环保产业，扶持发展循环经济，加强不可再生资源回收利用，吃干榨尽，变废为宝。推进电代煤、气代煤和节能改造，优先保障可再生能源发电上网。加快秸秆资源化利用。单位地区生产总值能耗降低15％，二氧化碳排放量降低18％，城市污水处理率达到95％。二是推进国土增绿全域化。全民绿化总动员，全市绿化强攻坚，增创更多生态财富，构筑可持续发展的绿色长城。森林覆盖率达到26％，城市绿化覆盖率达到45％。三是推进环境治理系统化。坚持点面结合、市县一体、综合施治，依法保护水生态，全面加强土治理，打好蓝天保卫战，让群众生活得清清爽爽，让良好生态成为推动发展最硬的软实力。

——围绕建设幸福大同，加快推动社会转型。坚持以人民为中心，按照习近平总书记的明确要求，把经济发展的成果更多地用于社会事业和民生改善上，兜住底线民生，保障基本民生，关注重点民生，打造幸福民生，努力实现：一是学有所教向学有优教转变。入园难问题彻底解决，普惠性幼儿园的比例不低于70％；义务教育“全面改薄”工作如期完成；城乡优质教育资源有序流动，基础教育办学质量明显提升；职教体系基本健全，产教融合度和地方经济相关性不断增强。二是劳有所得向劳有应得转变。加大支持双创力度，以创新带动创业，以创业带动就业，构筑新形势下劳动就业主渠道；落实政策、广开门路，为高校毕业生和广大就业人员开发新岗位；加强援助、托底帮扶，确保各类就业困难人员稳定就业。三是病有所医向病有良医转变。全民健身和全民健康深度融合，生命健康保障体系基本形成；医院专业水准和医生专业技术水平不断提升，三级医疗卫生服务体系更加健全；医保体系进一步完善，医改红利普惠人民群众。四是老有所养向老有颐养转变。养老保险体系全覆盖，养老保障水平逐步提高，养老事业和康养产业加速发展，尊老敬老氛围日益浓厚，社会养老服务规模和水平大幅提升。五是住有所居向住有宜居转变。住房保障体系进一步完善，城中村改造、危房改造、采煤沉陷区搬迁治理基本完成，城乡居住环境和管理服务不断改善。群众诉求表达、权益维护和社会保障机制趋于完善，帮扶济困常态化，社会救助亲情化，全面实现“困有所助”。凡是与老百姓密切相关的事情我们都要办好，“人民对美好生活的向往，就是我们的奋斗目标。”

三、2017年重点工作安排

今年是党和国家事业发展中具有重大意义的一年，也是我市新一届政府肩负重任、担当作为的起跑之年。政府工作要以习近平总书记系列重要讲话精神和治国理政新理念新思想新战略为指导，按照省委“一个指引、两手硬”重大思路和要求，深入贯彻落实省市党代会精神，坚持稳中求进总基调，推动全市经济社会持续健康发展。

今年经济社会发展主要指标是：地区生产总值增长5％左右；固定资产投资向注重质量和效益转变，完成省定任务；公共财政预算收入增长1．2％；社会消费品零售总额增长7％左右；城镇常住居民人均可支配收入增长6％左右；农村常住居民人均可支配收入增长6％以上。约束性指标完成省下达任务。

围绕上述目标，重点做好八个方面工作。

（一）稳定经济增长。突出抓好实体经济、县域经济和民营经济，以更加有力的举措，把控缓中趋稳，力促稳中向好，争取好中求快。

着力壮大实体经济。把推动现有工业增长作为稳增长的主要方面，精心组织，强化服务，确保企业生产经营正常运行。加快提升煤炭先进产能。着力培育新材料、磁动力、航空制造等中高端产业，促进工业经济新型化多元化。积极争取省财政技术改造专项资金，扶持中小企业做大做强，年内新增小升规企业11户。力争全市工业增长上半年转负为正，全年增长不低于3％。狠抓建筑业增量，征收房屋3万套，收储土地1万亩，适度扩大商品房建设规模，保持房地产业稳健发展，拉动建材、物流等相关产业，实现建筑业增加值增长5％以上。大力促进服务业发展，服务业增加值增长6．8％以上。支持和鼓励金融机构突出主业，重心下沉，服务实体经济。注重加强企业家队伍建设，提升企业家信心，充分发挥他们在稳增长中的主力军作用。

加快发展县域经济。立足资源禀赋、区位条件和产业基础，推动各县区发挥比较优势，尽快形成一至两个主导产业或发展方向，做大县域经济体量。左云侧重开发区发展、煤化工基地建设。浑源侧重景区开发、黄芪加工。灵丘侧重有机农业、红色旅游。广灵侧重杂粮加工、特色菌业。大同县侧重园区开发、黄花产业。天镇侧重功能农业、品牌劳务。阳高侧重康养产业、规模养殖。城区侧重古城开发、现代服务。矿区侧重产城融合、配套服务。南郊侧重综合能源、加工贸易、现代物流。新荣侧重新型材料、特色种养。

大力发展民营经济。鼓励支持民营企业、社会资本通过PPP模式进入基础设施、金融服务、公共事业等领域，激发市场活力。通过出资入股、股权收购、认购可转债、股权置换等多种方式，参与国有企业改制重组，实现合作共赢。发挥在同社会名流、名企业家的人脉资源，吸引优秀企业家来同创业。力争民营经济占比较去年提高2个百分点。

（二）加快产业升级。对接省产业转型六大工程，实施大同版八大工程，横向构筑产业群，纵向拉长产业链，发展多元化中高端现代产业体系。

培育战略性新兴产业。加强与中星微、浪潮、百度等信息技术企业和投资机构合作，争取建立大数据产业基地，大力发展数字经济。加快做大高铁受电弓、玄武岩纤维等前沿新材料产业。积极推进装配式绿色建筑集成产业基地、通用航空产业园、高效单晶光伏组件等项目的建设。支持发展煤制天然气、煤制烯烃等煤化工产品。

创新能源产业。严格控制煤炭产能总量，支持同煤创新发展。推进煤炭清洁高效利用。开工建设国家光伏示范基地二期、晋北风电基地项目。推进浑源抽水蓄能调峰电站和多能互补集成优化示范工程等项目前期。积极发展高载能产业，主攻电动汽车产业发展。

扶持绿色环保产业。加快环保产业绿色改造升级。推进煤矸石、粉煤灰、脱硫石膏等大宗工业固废综合利用。推广节能环保炉具和洁净煤使用。以塔山工业园区为试点，带动上下游企业绿色发展，开展绿色工业园区创建。建成建筑垃圾资源一体化项目。

改造提质传统优势产业。依托中车大同公司，发展电力机车和城市轨道交通装备制造。发挥军工企业技术优势，重点抓好重汽大同公司军用变速箱扩建、山柴煤层气发电机组推广应用项目。支持中银羊毛羊绒产业链生产基地等项目建设。做大做强医药产业，重点建设山西库邦年产80吨医药中间体、浑源正北芪开发等项目，打造全球最大抗生素生产基地和国内独具特色的中草药生产研发基地。

做强文化旅游产业。强化“旅游＋”意识，把握六字要诀，落实五个一批，创建全省文旅综改示范区，建成国内一流的旅游服务中心和中国大同旅游发展指数评价体系。加强文物保护，落实“三有三防”。重视古堡开发，包装边塞文化，发展长城旅游。依托古都灯会，弘扬年俗文化，打造“大同年”品牌。推广王府升锅宴，尽显大同老绝活，推动民间工艺产业化。大力发展冰雪和水上旅游产业，不断丰富和拓展冬令文化旅游，实现淡季不淡、旺季更旺。

发展现代服务业。完善配送网络，培育龙头企业。建成华阳国际陆港，抓紧建设晋北国际物流园。推进绿色金融创新，绿色发展基金投入运行，绿色债券发行和绿色银行筹办工作取得重要突破。

壮大特色现代农业。优先保障财政支农特别是农业县区支出，加强农业基础设施建设，鼓励农业科技创新，扶持龙头企业成长。大力推进农业供给侧结构性改革，以稳粮优经扩饲为方向，以做精杂粮、做优果菜、做强畜牧、做好药材为重点，构建大同黄花、阳高京杏、天镇小杂粮、新荣马铃薯、浑源黄芪、灵丘苦荞、广灵小米、左云肉羊八大特色农业发展区域，推动国家级种植与加工示范园区创建工作。支持发展功能农业，培育药食同源产品开发企业。推进农牧交错带核心示范区建设，实施10万亩苜蓿种植，抓好灵丘有机肉牛等基地建设，争创草畜一体化典型。提高主导农副产品深加工能力，增加绿色优质农产品供给。

“双创”孵化新产业新业态。建设中国国际技术转移大同合作中心、市中小企业创业基地和孵化基地，推动与中关村集团、清华科技园、农业科技城合作共建科技产业园。

（三）深化重点改革。以改革激活力，以开放强实力，以创新增动力，着力开创发展新局面。

深化重点领域和关键环节改革。深入推进“三去一降一补”。退出煤炭落后产能415万吨，抓紧完成棚改货币化安置任务，加大结构性减税力度，继续规范清理各类涉企收费，进一步降低企业生产经营成本。财政预算安排突出重点、有保有压，最大限度弥补经济建设和民生事业的各类短板。加快开发区改革创新发展。整合医药、装备制造、空港物流、新能源园区，完成大同经济技术开发区整合改制扩区调规工作，打造国家级综合性品牌示范园区，总规划面积达到239．4平方公里。支持和指导阳高、浑源、灵丘获批省级开发区。深化国企国资改革。经建投、经发投、金控集团组建运行，建立完善法人治理结构，增强企业竞争力。推进行政区划调整。方案批复后，迅速组织实施。支持同煤改革。成立市三供一业服务公司，完成首批职工家属区三供一业接收任务，启动其余办社会职能剥离工作。实施农村土地三权分置改革。基本完成农村土地承包经营权确权登记颁证，推动农村集体资产股份权能改革试点。推进农村土地经营权和住房财产权抵押贷款试点。采取股份合作、代耕代种等方式，加快大面积土地集中流转，实行规模化经营，促进农村土地资源优化配置。深化文化旅游体制改革。组建市和重点旅游县（区）旅发委，完成文旅集团组建并投入运营，实施云冈、恒山景区管理体制改革。推动电力体制改革。以工业园区为重点，多途径培育售电侧市场竞争主体，促进电力供需市场化。确保综合行政执法体制、统计管理体制、投融资体制、供销合作社等改革与全省同步推进。

全面提升对外开放水平。抢抓“一带一路”战略机遇，融入“中蒙俄”经济走廊，广泛开展项目洽谈、展会论坛以及各种经贸合作、文化交流等活动，进一步增强大同在区域协同发展中的地位和作用。加快大乌高铁项目前期，实现航空口岸正式开放。积极扩大出口规模。主动承接发达地区产业转移，推动京同区域深度合作。着力提高招商引资的精准度、实效性，重点考核项目的资金到位率和开工率。

大力实施创新驱动战略。落实企业技术创新“百项重点项目计划”，建成科技产业园一期，发挥创新引领作用。实施一批科技重点研发项目、应用示范工程，打通科技成果转化通道。大规模引进使用高学历高层次高素质人才，继续做好国家千人计划专家大同行工作；抓实院士工作站、专家工作站和创客中心创建工作；实施“凤凰计划”，促进“同才”回流。

（四）狠抓项目建设。强化项目倒逼机制，推动项目落地开工、投产见效。

突出重点抓项目。一是抓好字字百亿的16字项目。纳入重点工程范畴，保持定力，务求实效。加快建设投资170亿元的两河流域治理等工程，不断扩大投资量。抓紧开工抽水蓄能电站和超百亿康养等项目，尽快形成实物工作量。盯紧中海油煤制气等项目，不懈争取，力求突破。二是大力推进双环行动项目。以古城为心、以文瀛湖为肺、一方一圆谋划布局的27个项目，投资规模大，影响范围广，辐射带动强，必须抓紧抓实。潘家园文化商业项目建成运营，投资65亿元的古城东北隅开发工程加速推进，领阅综合体等一批总投资百亿元、总建筑面积300万平方米以上的房地产项目开工建设，投资10亿元的会展中心、投资36亿元的场馆、投资15．4亿元的古城南广场、投资8亿元的高铁广场等工程加快建设。卓达集团装配式建筑及城镇化、万龙冰雪旅游、凯帝斯电梯等项目加快落地。实施好北宇低速电动汽车等20个省重点工程、50个产业转型的市重点工程、21个县区重点工程项目。

整体推进抓项目。关注国家产业动向，紧盯我省投资重点，积极争取一批财政专项基金项目。主动承接产业转移，充实完善项目库，做好重大项目储备工作。巩固冬季行动战果，抓大项目，大抓项目，带动县区和部门谋划、招商、签约、落地、开工更多项目，实现项目建设全覆盖。

（五）推进脱贫攻坚。以脱贫攻坚统揽经济社会发展全局，把脱贫攻坚作为政治责任、头等大事和第一民生工程，在去年首战首胜的基础上，务求今年再战再胜，134个贫困村有序退出、6万人脱贫，南郊、新荣和左云三个区县插花贫困村全部脱贫。

落实精准方略。按照“六个精准”要求，实行政策向攻坚集成，资金向攻坚倾斜，项目向攻坚布局，人才向攻坚集聚，做到目标任务细化实化量化，政策措施到村到户到人。紧扣建档立卡关键环节，对贫困村、贫困户、贫困人口，实行定期核查、严格进退、动态管理，绝不搞数字脱贫、虚假脱贫，过好群众满意关，经得起实践和历史检验。

创新脱贫举措。分县实施、分类布点、分批推进、分年达标，确保八大工程二十个专项行动取得实效。通过争取专项资金、增加本级财政资金、整合使用涉农资金、撬动银行信贷资金等多种途径，帮助贫困户破解资金难题、发展项目。因地制宜发展一村一业，再建成一批光电村、旅游村、电商村，增强贫困村自身造血功能。按照“公路、城镇、园区”三靠近原则，把房子建起来，实施易地扶贫搬迁3．8万人。实施林业扶贫五个一批工程，优先保证贫困户退耕需求，优先选聘贫困户做生态护林员。加强教育扶贫，阻断贫困代际传递。量体裁衣、按需配菜，强化对贫困户子女职业技能免费培训，通过一技之长摆脱贫困。实行社保兜底脱贫，对脱贫线、低保线进行“两线合一”，防止困难群众因病因学因老返贫，做到应保尽保、特困特扶。

压实工作责任。对贫困县区脱贫攻坚进行单项考核，通过随机抽样、实地考核、引入第三方评价和社会综合评判的办法，夯实县区主体责任，调动工作积极性，确保各县区党政联手、扭住不放、攻坚到底。开展领导干部带头帮扶、机关单位包村帮扶、党员干部到户帮扶、工作队员驻村帮扶、第一书记任职帮扶“五帮联动”，做到贫困不除、队伍不散、工作不断。大力倡导社会扶贫，完善社会扶贫信息工作平台，开通“扶贫济困直通车”，让社会组织、爱心人士与贫困户“点对点”认亲、“面对面”帮扶，聚力脱贫攻坚，彰显社会温情。

（六）统筹城乡发展。加快中心城市、大县城、重点镇建设，更加注重品质提升，促进区域协调发展。

做好城市规划修编。加快新一轮总规、详规、专项规划修编。启动《大同中心城区总体城市设计》，从整体上引导城市新形象。开展古城、高铁站周边、老城与口泉结合部等区域城市空间规划利用研究，为下一步项目落地提供科学指导。编制海绵城市规划，提升我市生态系统功能。

优化城市空间布局。积极推进古城保护与开发，开工府衙修复，完成带状公园建设和护城河景观工程，改造完善老城区。加快御东新区建设，焕发城市生机，增强城市活力。完善口泉区设施配套，提升公共服务水平。深入推进大县城扩容提质，布局实施恒山等一批特色城镇，阳高、天镇等一批高铁小镇，抓好贫困村、采煤沉陷村、中心村、城中村和历史文化名村建设，申报绿色村庄216个，创建省市美丽宜居示范村15个。

加强基础设施建设。开工建设城市快速路、跨线立交桥、地下综合管廊，新建续建城市道路80公里，加快改造小街小巷。开源桥完工，汽车客运东站投入使用。推进综合客运枢纽建设，建成交通智能指挥控制中心。实施国道省道改建，开工建设古长城旅游路、东纵脱贫攻坚路，做好城市轻轨和王庄堡—阜平、大同南环高速公路前期工作。

提升城市管理水平。针对群众反映集中的领域和环节，制定详细方案，确定整改时限，明确责任单位，大力整治城市脏乱差，重拳整治营运车辆乱象，努力消除各种城市病，确保城市干净整洁、井然有序。创新城市管理方式，加快城市管理数字化、精细化和网格化，推动影响城市形象的问题及时发现、迅速会诊、有效解决。

（七）优化生态环境。不断强化绿水青山就是金山银山理念，持续加大生态环境治理力度，推进铁腕治污，深化综合整治，实行更加严格的考核办法和督查机制，营造山青水秀的生态环境。

改善水体质量。落实“河长制”，抓好桑干河等重点流域区域水污染和农业面源污染防治，加强河道巡查和企业监管，确保水质量明显好转，全市地表水水质优良断面比例达到16．7％，集中式饮用水水源地水质全部达到或优于国家三类标准。

强化土壤治理。开展土壤污染详查并分类治理。将土壤环境质量监测纳入例行环境监测体系，加强源头管控。加快矿山和非煤矿山生态修复，完成水保治理10万亩。启动混合（应急）垃圾填埋场、融雪剂溶化池建设，市区内建成10座垃圾中转站。

提升空气质量。坚决淘汰建成区燃煤锅炉，分步退出建成区原煤取暖，不断扩大洁净能源使用覆盖面。大力推进清洁能源供热工程。鼓励社会购买和使用环保节能汽车，依法淘汰黄标车、老旧车。加大建筑工地防尘管理，积极倡导文明施工。

坚持造林绿化。完成营造林24．5万亩，绿化村庄25个。加快儿童公园改造，启动大同公园改造和动物园迁建绿化，建设西京街绿轴景观带，完善御东公共活动走廊景观工程。

（八）做实民生工作。越是经济下行，越要高度关注和保障民生，这是我们义不容辞的责任。

发展社会事业。实施一批学校硬件建设工程，扎实推进义务教育均衡发展。支持办好大同大学。加快县级公立医院综合改革步伐，启动城市公立医院综合改革，为城乡居民提供家庭医生签约服务。加强生育医疗保健服务。继续推进全民参保。完善并兑现各类工资政策，努力增加人员工资收入。抓好各类政策性住房建设，开工城市棚户区改造31400套，改造农村危房21167户。

办好民生实事。新建社区公园8个。新开工建设便民市场10个。新建公交场站2处。新建公共停车场10个。新建人行天桥10座。在老城区、御东区、口泉区新增公共自行车1．5万辆。实施道路桥涵积水点排水改造工程。实施古城旱厕改造工程35处。组织开展千场大戏、万场电影文化惠民活动。新建农村老年人日间照料中心50个、城市社区服务中心20处。为就业大学生和引进人才提供1000套公租房。

维护安全稳定。坚持党政同责、一岗双责、企业主责、失职追责，铁的担当尽责、铁的手腕治患，铁的心肠问责，铁的办法治本。深入开展煤矿、非煤矿山、道路交通等领域安全生产隐患大排查大整治，坚决遏制重特大安全事故发生。创新社会治理方式，强化网格化管理，推进平安大同建设。高度重视社会矛盾化解，有效防范各种风险隐患，确保社会和谐稳定，扩大祥和共享新成果。

与此同时，继续落实全民健身计划，构建全民健身服务体系，提高人民健康水平。加强国防教育和国防动员建设，持续开展双拥工作，推进军民深度融合发展。做好修志编鉴工作，完成第二轮《大同市志》供稿，实现市县年鉴全覆盖。落实重大活动档案管理办法，发展档案事业。支持工会、共青团、妇联等人民团体更好地发挥桥梁纽带作用。做好外事、侨务、港澳、对台工作。加强民族宗教、防震减灾、对口援疆等工作。

各位代表，大同的历史需要340万人民共同创造，推动发展的重任需要我们共同承担。当前，大同正面临着经济下行困难期、各种风险矛盾交织期、发展转型升级关键期，既要求我们在攻坚克难中忠诚担当，也需要我们在继往开来中不负使命。今后五年，我们将恪尽职守，负重前行，咬定奋斗目标，铆紧各方责任，造实势、鼓实劲、办实事、求实效，与干部同舟共济，撸起袖子加油干；与人民同甘共苦，扑下身子抓落实，付出更多的心血，做出更大的努力，争取更好的结果！

各位代表，政府工作千头万绪，任务繁重，我们的每一次决策，都需要全市人民鼎力支持；每一项工作，都需要全市人民广泛参与；每一步跨越，都需要全市人民共同努力。新的一年，我们将更加崇尚法治、敬畏法律，认真执行人大及其常委会的各项决议决定，依法向人大及其常委会报告工作，积极接受人大的法律监督，自觉接受政协的民主监督，主动接受社会和舆论监督，着力加强审计监督，努力把政府的各项工作抓实抓好。我们将不断加强学习，苦练内功，做实底功，全面增强政府及其工作人员用好政策、推动发展、驾驭复杂局面的能力。我们将悉心听取民情，充分尊重民意，广泛汇集民智，努力把群众关注的事情办好。我们将主动转变政府职能，大力推进放管服效改革，落实好13710工作机制，服务基层、服务群众、服务发展。我们将认真落实全面从严治党总体要求和政府系统党风廉政建设主体责任，强化四个意识，严守《准则》《条例》，坚持执行八项规定，一以贯之纠正“四风”，开源节流过紧日子，着力塑造持廉守正的良好形象！

各位代表，过去的大同，曾经耀世辉煌；未来的大同，必将全面振兴；今天的大同，自当奋勇争先。让我们更加紧密地团结在以习近平同志为核心的党中央周围，在省委、省政府和市委的正确领导下，不忘初心，怀抱梦想，甩开膀子，奋力前行，为夺取全面建成小康社会的新胜利，为加快建设一个中心、三个大同，为创造更加美好灿烂的明天而努力奋斗，以优异成绩迎接党的十九大胜利召开！
